# Impacts of excision repair cross-complementing gene 1 (ERCC1), dihydropyrimidine dehydrogenase, and epidermal growth factor receptor on the outcomes of patients with advanced gastric cancer

**DOI:** 10.1038/sj.bjc.6604211

**Published:** 2008-01-29

**Authors:** J Matsubara, T Nishina, Y Yamada, T Moriwaki, T Shimoda, T Kajiwara, T E Nakajima, K Kato, T Hamaguchi, Y Shimada, Y Okayama, T Oka, K Shirao

**Affiliations:** 1 Gastrointestinal Oncology Division, National Cancer Center Hospital, 5-1-1 Tsukiji Chuo-ku, Tokyo 1040045, Japan; 2 Department of Gastroenterology, National Hospital Organization Shikoku Cancer Center, 160 Kou Minamiumemoto-cho, Matsuyama 7910280, Japan; 3 Clinical Laboratory Division, National Cancer Center Hospital, 5-1-1 Tsukiji Chuo-ku, Tokyo 1040045, Japan; 4 Personalized Medicine Research Laboratory, Taiho Pharmaceutical Co. Ltd, 224-2 Hiraishiebisuno Kawauchi-cho, Tokushima 7710194, Japan

**Keywords:** gastric cancer, excision repair cross-complementing gene 1, dihydropyrimidine dehydrogenase, epidermal growth factor receptor, prognostic factor

## Abstract

Using laser-captured microdissection and a real-time RT–PCR assay, we quantitatively evaluated mRNA levels of the following biomarkers in paraffin-embedded gastric cancer (GC) specimens obtained by surgical resection or biopsy: excision repair cross-complementing gene 1 (ERCC1), dihydropyrimidine dehydrogenase (DPD), methylenetetrahydrofolate reductase (MTHFR), epidermal growth factor receptor (EGFR), and five other biomarkers related to anticancer drug sensitivity. The study group comprised 140 patients who received first-line chemotherapy for advanced GC. All cancer specimens were obtained before chemotherapy. In patients who received first-line S-1 monotherapy (69 patients), low MTHFR expression correlated with a higher response rate (low: 44.9% *vs* high: 6.3%; *P*=0.006). In patients given first-line cisplatin-based regimens (combined with S-1 or irinotecan) (43 patients), low ERCC1 correlated with a higher response rate (low: 55.6% *vs* high: 18.8%; *P*=0.008). Multivariate survival analysis of all patients demonstrated that high ERCC1 (hazard ratio (HR): 2.38 (95% CI: 1.55–3.67)), high DPD (HR: 2.04 (1.37–3.02)), low EGFR (HR: 0.34 (0.20–0.56)), and an elevated serum alkaline phosphatase level (HR: 1.00 (1.001–1.002)) were significant predictors of poor survival. Our results suggest that these biomarkers are useful predictors of clinical outcomes in patients with advanced GC.

Gastric cancer (GC) is the second leading cause of cancer-related deaths worldwide, annually accounting for 40–50 deaths per 100 000 population in Japan and 5–15 deaths per 100 000 population in Europe (Parkin, 2001). During the past decade, newly developed cytotoxic drugs have been included in treatment regimens for GC. These new regimens have better response rates, often at the cost of higher incidences of severe adverse events (Ajani, 2005). This situation has created a greater need for diagnostic techniques that can predict clinical outcomes such as tumour response and survival in GC (Ichikawa, 2006). Considerable evidence suggests that the intratumour gene expressions of drug-metabolising enzymes, DNA repair enzymes, or angiogenic enzymes are useful predictors of treatment outcomes such as survival and the response to anticancer drugs (Backus *et al*, 2000; Ulrich *et al*, 2003; Marsh and McLeod, 2004). However, the clinical significance of these biomarkers remains unclear, especially in GC.

5-Fluorouracil (5-FU) and cisplatin are key drugs for the management of GC. Pharmacogenetic variability in metabolising enzymes of 5-FU and folate is a major determinant of the sensitivity to 5-FU and survival in GC (Lenz *et al*, 1996; Banerjee *et al*, 2002; Ichikawa *et al*, 2004; Napieralski *et al*, 2005). Several enzymes have key roles in the metabolic pathway of 5-FU and folate (Figure 1): thymidylate synthase (TS) is a target enzyme of 5-FU; dihydropyrimidine dehydrogenase (DPD) is a degrading enzyme of 5-FU; thymidine phosphorylase (TP) and orotate phosphoribosyl transferase (OPRT) are important metabolic enzymes; and dihydrofolate reductase (DHFR) and methylenetetrahydrofolate reductase (MTHFR) participate in folate metabolism. Lenz *et al* (1996) and Ichikawa *et al* (2004) have found that high TS mRNA expression in GC could predict poor clinical outcomes of treatment with 5-FU. Napieralski *et al* (2005) reported that high DPD expression in GC may correlate with poor survival and no response to 5-FU.

The cytotoxicity of cisplatin is attributed mainly to the induction of DNA intrastrand, interstrand, and DNA–protein crosslinks (Roberts and Thomson, 1979). Such DNA damage is thought to be repaired by the nucleotide excision pathway. Excision repair cross-complementing gene 1 (ERCC1) has a pivotal role in nucleotide excision repair and may promote the development of resistance to cisplatin (Dabholkar *et al*, 1992; Bramson and Panasci, 1993). Excision repair cross-complementing gene 1 is also associated with responses to cisplatin- and 5-FU-based chemotherapy in GC. Metzger *et al* (1998) reported that high ERCC1 expression in GC may be associated with poor survival and no response to cisplatin.

Other studies, however, have failed to confirm such correlations of the gene expressions of TS (Choi *et al*, 2001; Kwon *et al*, 2007), DPD (Ishikawa *et al*, 2000; Miyamoto *et al*, 2000), and ERCC1 (Napieralski *et al*, 2005) with the outcomes of chemotherapy. Further larger studies are thus required to confirm or refute previous claims.

This study was designed to further delineate the clinical implications of biomarkers and to identify potential predictors of the response to chemotherapy and survival in patients with GC. The epidermal growth factor receptor (EGFR) tyrosine kinase family and the vascular endothelial growth factor (VEGF) superfamily are also well-known mediators of tumour cell proliferation and tumour-related angiogenesis, which can influence tumour biology and survival (Carmeliet and Jain, 2000; Gamboa-Dominguez *et al*, 2004; Juttner *et al*, 2006). We tested the hypothesis that the clinical outcomes of chemotherapy (response rate, time to progression, and overall survival) in patients with advanced GC are related to the pretreatment intratumour mRNA levels of enzymes participating in critical pathways of drug resistance, such as 5-FU and folate metabolism (TS, DPD, TP, OPRT, DHFR, MTHFR), DNA repair (ERCC1), the EGFR signalling pathway (EGFR), and tumour-related angiogenesis (VEGF-A). We also compared the prognostic implications of these biomarkers with those of well-recognised prognostic factors (Chau *et al*, 2004; Lee *et al*, 2007).

## PATIENTS AND METHODS

### Patient eligibility

Patients with a diagnosis of histologically proven advanced GC were eligible for the study. Inclusion criteria were as follows: unresectable, locally-advanced, or metastatic disease; no prior chemotherapy and no prior adjuvant/neoadjuvant chemotherapy; specimens of primary gastric adenocarcinomas were obtained before the start of chemotherapy by surgical resection or biopsy at the National Cancer Center Hospital (Tokyo, Japan) or National Hospital Organization Shikoku Cancer Center (Matsuyama, Japan); first-line chemotherapy was received at either of the hospitals; radiographically measurable disease; and written informed consent. The tissue samples were collected retrospectively from patients who met these criteria. Measurable disease was assessed by computed tomography. Response was evaluated according to the standard UICC guidelines as complete response (CR), partial response (PR), no change (NC), or progressive disease (PD) (Hayward *et al*, 1978). Tumour response and survival times as of December 2006 were confirmed in all patients. The response rate was calculated as the ratio of (CR+PR)/(CR+PR+NC+PD). Written informed consent was obtained before treatment and evaluation of tumour samples. This study was approved by the institutional review boards of both hospitals.

### Clinical data

The following clinical data were included in analyses: performance status, liver and peritoneal metastases, and laboratory data at the start of chemotherapy, including leukocyte and lymphocyte counts and the serum levels of alkaline phosphatase (ALP), lactate dehydrogenase (LDH), albumin, C-reactive protein, and tumour markers (CEA, CA19-9).

### Chemotherapy

The following first-line chemotherapy regimens were administered to the patients in our study: S-1 monotherapy (*N*=69), cisplatin plus S-1 (*N*=14), cisplatin plus irinotecan (*N*=29), 5-FU monotherapy (*N*=23), and other regimens (5-FU plus methotrexate, *N*=2; paclitaxel, *N*=2; uracil/ftorafur, *N*=1). For S-1 monotherapy, patients received S-1 (40 mg m^−2^ twice daily) on days 1–28 of a 42-day cycle. Treatment with cisplatin plus S-1 consisted of cisplatin (60 mg m^−2^) on day 8 and S-1 (40 mg m^−2^ twice daily) on days 1–21 of a 35-day cycle. Treatment with cisplatin plus irinotecan consisted of cisplatin (80 mg m^−2^) on day 1 and irinotecan (70 mg m^−2^) on days 1 and 15 of a 28-day cycle. For 5-FU monotherapy, patients received 5-FU (800 mg m^−2^ day^−1^) as a continuous infusion on days 1–5 of a 28-day cycle.

### Laboratory methods

Ten-micrometre-thick sections obtained from identified areas with the highest tumour-cell concentration were mounted on uncoated glass slides. For histologic diagnosis, representative sections were stained with haematoxylin and eosin by standard methods. Before microdissection, sections were stained with nuclear fast red (American MasterTech Scientific, Lodi, CA, USA). The sections of interest were selectively isolated by laser-captured microdissection (PALM Microsystem, Leica, Wetzlar, Germany), according to standard procedures (Bonner *et al*, 1997). The dissected particles of tissue were transferred to a reaction tube containing 400 *μ*l of RNA lysis buffer.

The samples were homogenised and heated at 92°C for 30 min. Fifty microlitres of 2 M sodium acetate was added at pH 4.0, followed by 600 *μ*l of freshly prepared phenol/chloroform/isoamyl alcohol (250 : 50 : 1). The tubes were placed on ice for 15 min and then centrifuged at 13 000 r.p.m. for 8 min in a chilled (8°C) centrifuge. The upper aqueous phase was carefully removed. Glycogen (10 *μ*l) and 300–400 *μ*l of isopropanol were added. The tubes were chilled at −20°C for 30–45 min to precipitate the RNA. The samples were washed in 500 *μ*l of 75% ethanol and air-dried for 15 min. The pellet was resuspended in 50 *μ*l of 5 mM Tris. Finally, cDNA was prepared as described by Lord and colleagues (Lord *et al*, 2000).

Quantification of nine genes of interest and an internal reference gene (*β*-actin) was performed with a fluorescence-based real-time detection method (ABI PRISM 7900 Sequence Detection System, TaqMan®, Perkin-Elmer (PE) Applied Biosystem, Foster City, CA, USA) using the standard curve method. The PCR reaction mixture consisted of 1200 nM of each primer, 200 nM of probe, 0.4 U of AmpliTaq gold polymerase, 200 nM each of dATP, dCTP, dGTP, and dTTP, 3.5 mM of MgCl_2_, and 1 × Taqman buffer A containing a reference dye. The final volume of the reaction mixture was 20 *μ*l (all reagents from PE Applied Biosystems, Foster City, CA, USA). Cycling conditions were 50°C for 2 min and 95°C for 10 min, followed by 46 cycles of 95°C for 15 s and 60°C for 1 min. The primers and probes used are listed in Table 1. Gene expression values (relative mRNA levels) are expressed as ratios (differences between *C*_t_ values) between the gene of interest and an internal reference gene (*β*-actin).

For each gene, we establish a usable *C*_t_ range for the data and document the precision of the measurements within the usable range. For maximum accuracy, we demonstrate that the slopes of the plots of Δ*C*_t_ *vs* Log pg. RNA for target genes and the housekeeping gene (actin) demonstrate parallelism. Each replicate *C*_t_ data point is the average of *C*_t_ values obtained in three PCR reactions. To compare the results of two different TaqMan plates with each other, the same standardised samples are analysed on every plate.

### Statistical analysis

We examined the objective tumour response to chemotherapy, time to progression, and overall survival. Time to progression and overall survival were calculated as the period from the start of first-line chemotherapy until disease progression or death from any cause, respectively. If patients were lost to follow-up, data were censored at the date of the last evaluation.

To assess associations of gene expression levels with tumour response, time to progression, and overall survival, the expression levels of each gene were categorised into low and high values at optimal cutoff points. The maximal *χ*^2^ method (Halpern, 1982; Miller and Siegmund, 1982; Lausen and Schumacher, 1992) was used to determine which gene expression (optimal cutoff point) best segregated patients into poor- and good-outcome subgroups (in terms of likelihood of response and survival). To determine the corrected *P*-values on the basis of the maximal *χ*^2^ analysis, 2000 bootstrap-like simulations were used in univariate analyses to estimate the distribution of the maximal *χ*^2^ statistics under the null hypothesis of no association. The clinical laboratory data were treated as continuous variables. The estimates of hazard ratios (HRs) with 95% CIs, on the basis of a Cox proportional hazards model, were used to provide quantitative summaries of the gene expression data.

All reported *P*-values are two-sided, and the level of statistical significance was set at *P*<0.010. Variables for multivariate analysis were selected by the Stepwise Method, using a significance level of <0.010 for entering into or remaining in the model. All analyses were performed using the statistical software package R, version 2.4.1, and the SAS statistical package, version 9.1.3 (SAS Institute Inc., Cary, NC, USA).

## RESULTS

A total of 140 patients were eligible for the study. Eighty-six patients (61%) were recruited at the National Cancer Center Hospital and 54 patients (39%) at the National Hospital Organization Shikoku Cancer Center. Chemotherapy began in July 1997 in the first patient and in June 2004 in the last patient. The demographic characteristics of the patients are shown in Table 2. There were 108 (77%) men and 32 (23%) women with a median age of 65 years. At the time of analysis, 131 (94%) patients had died and nine (6%) patients were alive.

The chemotherapy regimens received by the patients and the response rates are also listed in Table 2. Many patients received S-1 monotherapy or cisplatin-based regimens as first-line treatment. The response rates with first-line chemotherapies in our study were comparable to those reported previously (Sakata *et al*, 1998; Boku *et al*, 1999; Ohtsu *et al*, 2003; Ajani *et al*, 2006).

### Gene expression levels of selected biomarkers, clinical data, and overall survival in all patients

Gene expression levels of selected biomarkers were quantifiable in 88.6–99.3% of the 140 tumours (Table 3). Gene expression cutoff values in terms of overall survival were defined by using the maximal *χ*^2^ method, and corrected *P*-values were calculated for each single gene. On univariate analyses, overall survival in the study group as a whole correlated with the expression levels of ERCC1, DPD, EGFR, and TS, the serum levels of LDH and ALP, and performance status (Table 4). Using these significant mRNA factors on univariate analyses, we performed combined analysis. Patients with low mRNA expressions of ERCC1, DPD, TS, and high expression of EGFR (*N*=30) had significantly longer overall survival than did the other patients (*N*=106) (median overall survival, 22.0 *vs* 11.2 months; *P*<0.001, log-rank test; Figure 2). Multivariate analysis with a Cox proportional hazards model demonstrated that high ERCC1 expression (HR: 2.38 (1.55–3.67)), high DPD expression (HR: 2.04 (1.37–3.02)), low EGFR expression (HR: 0.34 (0.20–0.56)), and an elevated serum ALP level (HR: 1.00 (1.001–1.002)) were significant predictors of poor survival (Table 4).

### Gene expression levels of selected biomarkers, tumour response, and time to progression in patients treated with S-1 monotherapy or cisplatin-based regimens as first-line chemotherapy

To better understand the relation between mRNA levels of selected biomarkers and treatment outcomes with each chemotherapy regimen, we performed subgroup analyses. Gene expression cutoff values that best segregated patients into poor- and good-response subgroups were defined by using the maximal *χ*^2^ method. In patients given first-line S-1 monotherapy, low MTHFR (low: 44.9% *vs* high: 6.3%, *P*=0.006) gene expression alone correlated with a better response (Table 5). Expressions of the other eight genes did not correlate with response. In patients treated with first-line cisplatin-based regimens (combined with S-1 or irinotecan), low ERCC1 (low: 55.6% *vs* high: 18.8%, *P*=0.008) gene expression alone correlated with a better response (Table 5). Expressions of the other eight genes did not show any correlation with response.

Gene expression cutoff values and the corrected *P*-values for time to progression analyses were determined by the same methods as those used in the analyses of overall survival. In patients given first-line S-1 monotherapy, expression levels of DHFR and EGFR were significantly associated with the time to progression (Table 6). When 2.89 × 10^−3^ was used as the cutoff value for DHFR, the median time to progression was 6.1 months in the low-expression group and 4.0 months in the high-expression group (corrected log-rank *P*=0.003, HR: 2.43 (95% CI: 1.37–4.29)). DHFR gene expression correlated with TS expression, with a Spearman's rank correlation coefficient of 0.456 (*P*<0.001). When a cutoff value of 0.33 × 10^−3^ was used for EGFR, the median time to progression was significantly longer in the high EGFR expression group (low: 2.8 months *vs* high: 5.3 months, *P*=0.007, HR: 0.31 (0.16–0.62)). The association between expression levels of TS, DPD, TP, OPRT, MTHFR, ERCC1, and VEGF-A and the time to progression did not show significant results (Table 6).

In patients who received cisplatin-based regimens as first-line chemotherapy, expression levels of DPD and MTHFR correlated with the time to progression (Table 6). At a DPD cutoff value of 1.55 × 10^−3^, the median time to progression was 4.6 months in the low DPD expression group as compared with only 1.2 months in the high DPD expression group (*P*=0.008, HR: 4.87 (1.75–13.53)). At a MTHFR cutoff value of 0.94 × 10^−3^, the median time to progression was significantly longer in the high-expression group (low: 2.9 months *vs* high: 5.9 months, *P*=0.007, HR: 0.17 (0.07–0.42)). The association between expression levels of the other seven genes and time to progression did not show significant results (Table 6).

## DISCUSSION

In this study, we analysed mRNA expression levels of nine genes involved in 5-FU and folate metabolism, DNA repair, and angiogenesis in primary tumours from 140 patients with advanced GC. Our goal was to determine whether such expression levels are related to treatment outcomes such as survival and response. We found that high DPD expression, high ERCC1 expression, and low EGFR expression in GC specimens were significant predictors of poor survival in advanced GC. Recently, several studies have reported that patients’ genetic profiles are related to the outcomes of cancer therapy (van ‘t Veer *et al*, 2002; Ruzzo *et al*, 2006). In colorectal cancer, since many studies have examined molecular predictors of outcomes during the past decade, TS, DPD, and TP were newly included in ‘ASCO 2006 tumour marker guidelines in gastrointestinal cancer’ (Locker *et al*, 2006). Because sufficient supporting evidence is lacking, however, the guidelines recommend that these biomarkers should not yet be used clinically to predict prognosis or treatment response. Further studies are therefore needed to more clearly define the relation between mRNA expression levels and clinical outcomes.

Our study showed that gene expression levels of DPD (related to the pharmacokinetics of fluoropyrimidines) and ERCC1 (related to the pharmacodynamics of cisplatin) had significant impacts on the overall survival of patients with advanced GC. This finding is consistent with the results of previous investigations (Metzger *et al*, 1998; Terashima *et al*, 2002; Napieralski *et al*, 2005). S-1, an oral DPD inhibitory fluoropyrimidine, is a novel antitumour drug combining tegafur (FT: a prodrug of 5-FU), gimeracil (CDHP: 5-chloro-2,4 dihydropyridine), and oteracil (Oxo: potassium oxonate) (Shirasaka *et al*, 1993, 1996). CDHP inhibits DPD activity and therefore prevents fluoropyrimidine degradation (Shirasaka *et al*, 1996). Oxo is a gastrointestinal tract adverse effect modulator (Shirasaka *et al*, 1993). In Japan, S-1 as monotherapy or combined with cisplatin is a standard regimen for advanced GC (Sakata *et al*, 1998; Ajani *et al*, 2006). Boku *et al* (2007) reported the result of a randomised controlled trial showing that S-1 is a promising standard regimen as compared with 5-FU, and Narahara *et al* (2007) showed that S-1 plus cisplatin is superior to S-1 alone. A multinational phase III study comparing S-1 plus cisplatin with 5-FU plus cisplatin (control regimen) is now underway. In the future, S-1 combined with cisplatin may become a standard regimen not only in Japan but also worldwide. In our study, 129 patients (92%) received S-1- or 5-FU-based regimens, and 73 patients (52%) received cisplatin-based regimens as first-line or subsequent chemotherapy. Our results strongly suggest that tumours with high DPD and ERCC1 gene expression are unlikely to respond to current standard therapy, resulting in inadequate tumour control and poor outcomes. Patients with such tumours would require newly developed drugs and combined treatment modalities tailored to their specific needs.

**Figure 1:**
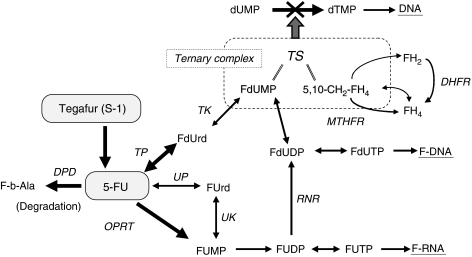
5-Fluorouracil and folate metabolic pathways. Genes examined in our study are shown in bold. DPD, dihydropyrimidine dehydrogenase; DHFR, dihydrofolate reductase; MTHFR, methylenetetrahydrofolate reductase; OPRT, orotate phosphoribosyl transferase; TS, thymidylate synthase; TP, thymidine phosphorylase; . The official Human Genome Organization gene nomenclature is used. Common or alternative names for each gene can be found at http://pharmacogenetics.wustl. edu.

**Figure 2:**
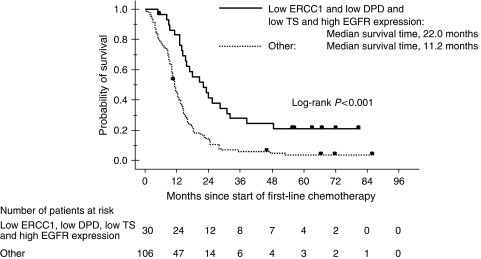
Kaplan–Meier plot of overall survival for all patients according to ERCC1, DPD, TS, and EGFR mRNA expression levels.

Patients with low DHFR expression had a higher response rate and a longer time to progression while receiving S-1 monotherapy (Tables 5 and 6). DHFR is a key enzyme of folate metabolism. DHFR converts intracellular inactive dihydrofolate back to active tetrahydrofolate, which is reused in deoxythymidine-5′-monophosphate synthesis (Figure 1) and is crucial for 5-FU antitumour activity. Sowers *et al* (2003) reported that E2F transcription factors may participate in the regulation of both TS and DHFR expression. We showed that a Spearman's correlation coefficient for TS/DHFR was 0.456 (*P*<0.001). Backus *et al* (2000) reported that low TS expression *in vitro* correlated with increased sensitivity to 5-FU. Several clinical studies have found that patients with low TS gene expression in primary GC correlate with a better tumour response and longer survival after 5-FU or S-1 treatment (Lenz *et al*, 1996; Ichikawa *et al*, 2004). The results of these studies suggest that low DHFR expression is associated with better clinical outcomes in patients given S-1 monotherapy, consistent with the results of our study. DHFR might thus be a candidate predictive biomarker of the response to S-1 treatment. Our data suggested that low DHFR expression may be an important determinant of tumour-cell sensitivity to S-1.

In patients who received S-1 monotherapy, low MTHFR gene expression also correlated with a better tumour response (Table 5). Some studies have reported that the *MTHFR 677T* mutation, linked to the reduced activity of MTHFR, increases chemosensitivity to 5-FU (Cohen *et al*, 2003; Sohn *et al*, 2004), whereas others have had inconsistent results (Etienne *et al*, 2004; Ruzzo *et al*, 2006). Although MTHFR and DHFR are key enzymes in folate metabolism, the role of MTHFR gene expression in the antitumour activity of 5-FU remains controversial.

In patients given cisplatin-based regimens as first-line chemotherapy, low ERCC1 mRNA expression alone correlated with a better tumour response, confirming a previously reported association (Metzger *et al*, 1998). With respect to EGFR gene expression, evidence supporting a correlation between mRNA expression levels and survival or time to progression in GC is scant. Vallbohmer *et al* (2006) reported that high mRNA expression of EGFR was associated with a better response as well as longer progression-free and overall survival in patients with colorectal cancer who received irinotecan therapy, which is partially in accord with our findings. In contrast, Gamboa-Dominguez *et al* (2004) found that strong membranous staining of EGFR on immunohistochemical analysis correlated with poor survival. The clinical implications of EGFR gene expression thus remain controversial.

In conclusion, our study provides evidence that high DPD, high ERCC1, and low EGFR gene expression levels in GC specimens and an elevated serum ALP level are risk factors for poor survival in patients with advanced GC. To the best of our knowledge, this is the first study showing that mRNA expression levels of molecular markers in primary GC had as much impact on survival outcomes as did well-recognised prognostic factors. The results of our analysis will hopefully provide a more rational basis for clinical decision-making, risk stratification of patients, and selection of management strategies as well as suggest benchmarks for future randomised controlled trials. Our relatively small sample size precludes drawing any firm conclusions, and candidate biomarkers must be validated in prospective studies. To confirm and extend the results of this exploratory study, larger studies are being planned in Japan.

## Figures and Tables

**Table 1:** Primer and probe sequences for quantitative RT–PCR

**Gene**	**Forward primer (5′-3′)**	**Reverse primer (5′-3′)**	**Taqman® probe (5′-3′)**
TS	GCCTCGGTGTGCCTTTCA	CCCGTGATGTGCGCAAT	TCGCCAGCTACGCCCTGCTCA
DPD	AGGACGCAAGGAGGGTTTG	GTCCGCCGAGTCCTTACTGA	CAGTGCCTACAGTCTCGAGTCTGCCAGTG
TP	CCTGCGGACGGAATCCT	GCTGTGATGAGTGGCAGGCT	CAGCCAGAGATGTGACAGCCACCGT
OPRT	TAGTGTTTTGGAAACTGTTGAGGTT	CTTGCCTCCCTGCTCTCTGT	TGGCATCAGTGACCTTCAAGCCCTCCT
DHFR	GTCCTCCCGCTGCTGTCA	GCCGATGCCCATGTTCTG	TTCGCTAAACTGCATCGTCGCTGTGTC
MTHFR	CGGGTTAATTACCACCTTGTCAA	GCATTCGGCTGCAGTTCA	TGAAGGGTGAAAACATCACCAATGCCC
ERCC1	GGGAATTTGGCGACGTAATTC	GCGGAGGCTGAGGAACAG	CACAGGTGCTCTGGCCCAGCACATA
EGFR	TGCGTCTCTTGCCGGAAT	GGCTCACCCTCCAGAAGGTT	ACGCATTCCCTGCCTCGGCTG
VEGF-A	AGTGGTCCCAGGCTGCAC	TCCATGAACTTCACCACTTCGT	TGATTCTGCCCTCCTCCTTCTGCCAT
*β*-Actin	GAGCGCGGCTACAGCTT	TCCTTAATGTCACGCACGATTT	ACCACCACGGCCGAGCGG

Abbreviations: DHFR=dihydrofolate reductase; DPD=dihydropyrimidine dehydrogenase; EGFR=epidermal growth factor receptor; ERCC1=excision repair cross-complementing gene 1; MTHFR=methylenetetrahydrofolate reductase; OPRT=orotate phosphoribosyl transferase; TP=thymidine phosphorylase; TS=thymidylate synthase; VEGF-A=vascular endothelial growth factor-A.

**Table 2:** Patient characteristics

	**Patients**	
**Characteristic**	**No.**	**%**
All patients	140	
*Sex*		
Male	108	77
Female	32	23
		
*Age (years)*		
Median	65	
Range	18–87	
		
*ECOG performance status*		
0	70	50
1	62	44
2	8	6
		
*Metastatic site*		
Lymph nodes	87	62
Peritoneum	43	31
Liver	43	31
Lung	8	6
Other	9	6
		
*Histological type*		
Intestinal	60	43
Diffuse	80	57
		
*First-line chemotherapy regimen*		*(Response rate*^a^ *(95% CI))*
S-1	69	34.8 (23.7–47.2)
Cisplatin+S-1	14	35.7 (12.8–64.9)
Cisplatin+irinotecan	29	44.8 (26.5–64.3)
5-FU	23	4.3 (0.1–22.0)
5-FU+methotrexate	2	0
Paclitaxel	2	50.0 (1.3–98.7)
Uracil/ftorafur (UFT®)	1	0

Abbreviation: ECOG=Eastern Cooperative Oncology Group.

a Response rate was calculated as the ratio of (CR+PR)/(CR+PR+NC+PD).

**Table 3:** Gene expression levels of analysed biomarkers in all 140 patients

		**mRNA expression levels relative to *β*-actin ( × 10^−3^)**	
**Gene**	**No. of patients (%)**	**Median**	**Range**
TS	139 (99.3)	2.81	0.84–16.05
DPD	134 (95.7)	0.85	0.07–13.54
TP	139 (99.3)	5.96	0.82–32.01
OPRT	138 (98.6)	0.99	0.28–4.55
DHFR	124 (88.6)	2.94	0.42–8.69
MTHFR	136 (97.1)	1.24	0.25–8.20
ERCC1	139 (99.3)	1.03	0.22–6.22
EGFR	126 (90.0)	1.24	0.12–57.78
VEGF-A	137 (97.9)	4.89	1.07–30.23

Abbreviations: DHFR=dihydrofolate reductase; DPD=dihydropyrimidine dehydrogenase; EGFR=epidermal growth factor receptor; ERCC1=excision repair cross-complementing gene 1; MTHFR=methylenetetrahydrofolate reductase; OPRT=orotate phosphoribosyl transferase; TP=thymidine phosphorylase; TS=thymidylate synthase; VEGF-A=vascular endothelial growth factor-A.

**Table 4:** Univariate analysis and Cox regression multivariate analysis of overall survival in all patients included in this study: correlation with mRNA expression levels and clinical data

				**Univariate analysis**		**Multivariate analysis**	
**Factor^a^**	**Cut point**	**No. of patients**	**Median (months)**	**Hazard ratio (95% CI)**	***P***	**Hazard ratio (95% CI)**	***P***
LDH	Continuous	—	—	—	<0.001		
	Variable			1.00 (1.000–1.001)			
ALP	Continuous	—	—	—	<0.001	—	< 0.001
	Variable			1.00 (1.001–1.002)		1.00 (1.001–1.002)	
ERCC1	⩽1.42 × 10^−3^	103	14.3	1	0.002	1	< 0.001
	>1.42 × 10^−3^	36	9.8	2.12 (1.41–3.18)		2.38 (1.55–3.67)	
DPD	⩽1.18 × 10^−3^	93	14.5	1	0.003	1	< 0.001
	>1.18 × 10^−3^	44	10.2	1.95 (1.34–2.83)		2.04 (1.37–3.02)	
PS	Continuous	—	—	—	0.004		
	Variable			1.55 (1.15–2.08)			
EGFR	⩽0.33 × 10^−3^	21	8.2	1	0.005	1	< 0.001
	>0.33 × 10^−3^	118	13.6	0.42 (0.26–0.69)		0.34 (0.20–0.56)	
TS	⩽2.61 × 10^−3^	62	16.0	1	0.010		
	>2.61 × 10^−3^	77	11.2	1.64 (1.15–2.34)			

Abbreviations: ALP=alkaline phosphatase; DPD=dihydropyrimidine dehydrogenase; EGFR=epidermal growth factor receptor; ERCC1=excision repair cross-complementing gene 1; LDH=lactate dehydrogenase; PS=performance status; TS=thymidylate synthase.

Note: ‘Cutoff point’ for mRNA expression level was determined by the maximal *χ*^2^ method.

a Factors with *P*-values of <0.010 in univariate analyses are listed in ascending order of *P*-values. The stepwise method was used to select factors for multivariate analysis.

**Table 5:** Gene expression levels and tumour response in patients with advanced gastric cancer according to first-line chemotherapy

	**S-1 monotherapy (*N*=69)**					**Cisplatin-based regimens^a^ (*N*=43)**				
**Factor**	**Total no. of patients**	**Cut point ( × 10^−3^)**	**RR (%) in low group**	**RR (%) in high group**	***P***	**Total no. of patients**	**Cut point ( × 10^−3^)**	**RR (%) in low group**	**RR (%) in high group**	***P***
TS	66	3.67	45.2 (19/42)	20.8 (5/24)	0.044	43	3.43	50.0 (15/30)	23.1 (3/13)	0.103
DPD	65	0.83	25.9 (7/27)	44.7 (17/38)	0.119	42	0.84	28.0 (7/25)	58.8 (10/17)	0.041
TP	66	5.37	25.9 (7/27)	43.6 (17/39)	0.121	43	7.81	32.1 (9/28)	60.0 (9/15)	0.049
OPRT	65	0.61	0 (0/6)	39.0 (23/59)	0.059	43	0.94	57.1 (12/21)	27.3 (6/22)	0.029
DHFR	59	1.64	57.1 (4/7)	28.8 (15/52)	0.105	39	2.32	31.6 (6/19)	45.0 (9/20)	0.323
MTHFR	65	1.82	**44.9** (**22/49)**	**6.3** (**1/16)**	**0.006**	43	1.15	52.2 (12/23)	30.0 (6/20)	0.152
ERCC1	65	0.92	50.0 (14/28)	24.3 (9/37)	0.033	43	1.18	**55.6** (**15/27)**	**18.8** (**3/16)**	**0.008**
EGFR	66	1.20	45.7 (16/35)	25.8 (8/31)	0.094	43	1.39	51.7 (15/29)	21.4 (3/14)	0.049
VEGF-A	65	2.70	54.5 (6/11)	31.5 (17/54)	0.104	43	6.52	53.8 (14/26)	23.5 (4/17)	0.022

Abbreviations: DHFR=dihydrofolate reductase; DPD=dihydropyrimidine dehydrogenase; EGFR=epidermal growth factor receptor; ERCC1=excision repair cross-complementing gene 1; MTHFR=methylenetetrahydrofolate reductase; OPRT=orotate phosphoribosyl transferase; RR=response rate; TP=thymidine phosphorylase; TS=thymidylate synthase; VEGF-A=vascular endothelial growth factor-A.

Note: ‘Cutoff point’ was determined by the maximal *χ*^2^ method. The level of significance was set at *P*<0.010. Significant values are shown in bold.

a Cisplatin-based regimens: cisplatin+S-1 and cisplatin+irinotecan.

**Table 6:** Univariate analyses of time to progression in patients with advanced gastric cancer treated with S-1 monotherapy or cisplatin-based regimens as first-line chemotherapy: correlation with mRNA expression levels

	**S-1 monotherapy (*N*=69)**					**Cisplatin-based regimens^a^ (*N*=43)**				
**Factor**	**Cut point ( × 10^−3^)**	**No. of patients**	**Median (months)**	**Hazard ratio** (**95% CI)**	***P***	**Cut point ( × 10^−3^)**	**No. of patients**	**Median (months)**	**Hazard ratio** (**95% CI)**	***P***
TS	⩽5.27	60	4.5	1	0.131	⩽3.36	29	5.4	1	0.140
	>5.27	9	4.2	2.11 (0.97–4.55)		>3.36	14	3.9	1.68 (0.87–3.24)	
DPD	⩽1.57	51	4.9	1	0.080	⩽1.55	37	4.6	1	**0.008**
	>1.57	17	4.0	1.90 (1.06–3.43)		>1.55	5	1.2	4.87 (1.75–13.53)	
TP	⩽5.58	31	4.0	1	0.207	⩽8.31	30	4.2	1	0.222
	>5.58	38	5.1	0.72 (0.44–1.18)		>8.31	13	6.2	0.62 (0.32–1.22)	
OPRT	⩽1.44	48	4.2	1	0.223	⩽0.92	20	6.2	1	0.036
	>1.44	20	4.2	1.37 (0.79–2.36)		>0.92	23	3.9	1.93 (1.02–3.66)	
DHFR	⩽2.89	29	6.1	1	0.003	⩽5.82	35	4.5	1	0.215
	>2.89	33	4.0	2.43 (1.37–4.29)		>5.82	4	14.6	0.41 (0.12–1.39)	
MTHFR	⩽1.04	20	2.9	1	0.158	⩽0.94	10	2.9	1	**0.001**
	>1.04	48	5.1	0.59 (0.34–1.01)		>0.94	33	5.9	0.17 (0.07–0.42)	
ERCC1	⩽1.30	49	4.2	1	0.370	⩽1.12	24	4.2	1	0.318
	>1.30	19	4.4	0.72 (0.41–1.27)		>1.12	19	5.9	0.75 (0.41–1.39)	
EGFR	⩽0.33	12	2.8	1	**0.007**	⩽0.81	16	3.9	1	0.158
	>0.33	57	5.3	0.31 (0.16–0.62)		>0.81	27	5.2	0.53 (0.28–1.03)	
VEGF-A	⩽2.45	7	1.9	1	0.193	⩽7.86	34	3.8	1	0.130
	>2.45	61	4.4	0.46 (0.21–1.02)		>7.86	9	7.2	0.43 (0.20–0.93)	

Abbreviations: DHFR=dihydrofolate reductase; DPD=dihydropyrimidine dehydrogenase; EGFR=epidermal growth factor receptor; ERCC1=excision repair cross-complementing gene 1; MTHFR=methylenetetrahydrofolate reductase; OPRT=orotate phosphoribosyl transferase; TP=thymidine phosphorylase; TS=thymidylate synthase; VEGF-A=vascular endothelial growth factor-A.

Note: ‘Cutoff point’ was determined by the maximal *χ*^2^ method. The level of significance was set at *P*<0.010. Significant values are shown in bold.

a Cisplatin-based regimens: cisplatin+S-1 and cisplatin+irinotecan.
